# Laparoscopic Management of Abdominal Apoplexy

**DOI:** 10.7759/cureus.4324

**Published:** 2019-03-26

**Authors:** Yang Hwang, Richard Gartrell, Nicole Winter, Harsheet Sethi, Chung Kwun Won

**Affiliations:** 1 Department of Surgery, Redland Alexandra Hospital, Brisbane, AUS; 2 Department of Surgery, Princess Alexandra Hospital, Brisbane, AUS

**Keywords:** haematoma, spontaneous, abdominal, apoplexy, intraabdominal, intraperitoneal, hemorrhage, idiopathic, laparoscopic, minimally invasive

## Abstract

Abdominal apoplexy, otherwise known as intraperitoneal idiopathic spontaneous haemorrhage, is a rare condition that presents as a diagnostic dilemma and is associated with high mortality. Symptoms and signs typically are similar to other conditions presenting with upper abdominal peritonitis. Intraabdominal haemorrhage can occur from many different causes, including trauma, iatrogenic, ruptured aneurysm, gynaecological conditions, malignancy, and inflammatory or autoimmune processes. Spontaneous or idiopathic causes are much rarer. Prompt diagnosis and ligation of the bleeding vessel usually result in a good outcome. Most cases described involve males in the fifth and sixth decade of life who present in the setting of hypertension and known atherosclerotic disease and are managed with laparotomy or are diagnosed at autopsy. We present a case of abdominal apoplexy managed laparoscopically in a healthy 20-year-old male with no pre-existing medical conditions. This case highlights the importance to consider abdominal apoplexy in any demographic.

## Introduction

Abdominal apoplexy, otherwise known as intraperitoneal idiopathic spontaneous haemorrhage, is a rare condition that usually presents as a diagnostic dilemma and is associated with high mortality. Symptoms and signs typically are similar to other conditions presenting with upper abdominal peritonitis. The term abdominal apoplexy was first used in 1931 by Green and Powers to describe a case series of spontaneous haemoperitoneum resulting from the spontaneous rupture of an intraabdominal vessel [1]. Since it was first described, less than 200 cases have been reported in the English literature [2]. The patient usually presents with abdominal pain mimicking pancreatitis or perforated peptic ulcer disease and haemodynamic instability. Massive haemoperitoneums of over 5-litres have been found on laparotomy or autopsy. Patients usually have a good outcome after surgery and ligation of the bleeding vessel, although, in 20% - 30% of cases, the source is not located [2].

## Case presentation

**Figure 1:**
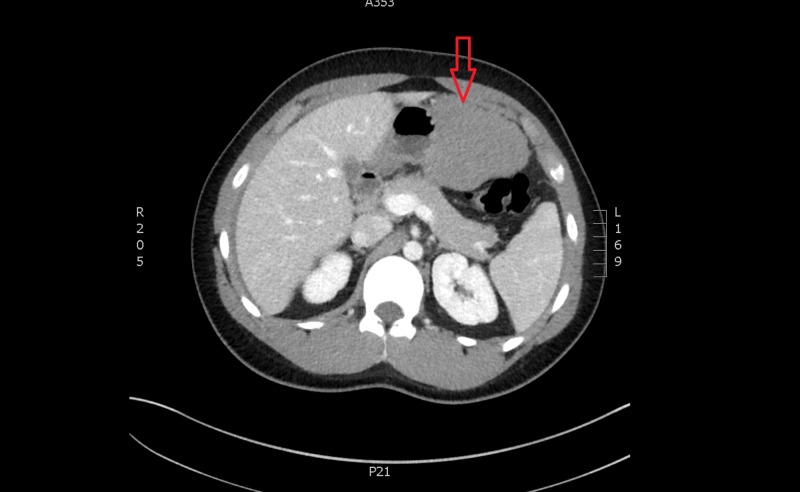
Computed tomography of the abdomen (axial) showing a heterogeneous mass from the greater curvature of the stomach (see red arrow)

**Figure 2:**
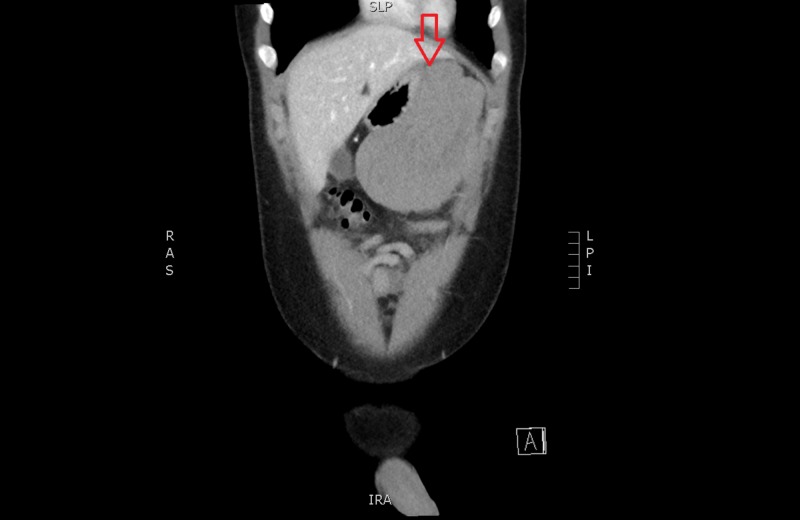
Computed tomography of the abdomen (coronal) showing a heterogeneous mass from the greater curvature of the stomach (see red arrow)

**Figure 3:**
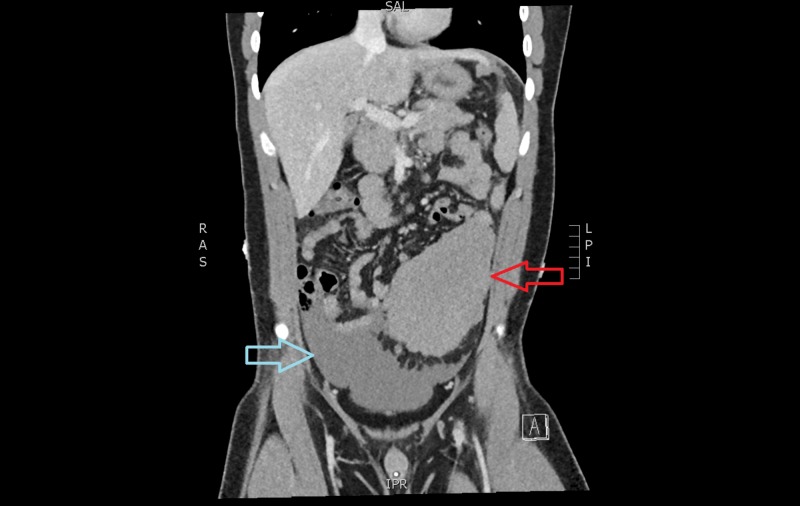
Computed tomographic angiogram (in venous phase) showing caudal migration of the heterogeneous mass and increased haemoperitoneum Red arrow: caudal migration of heterogeneous mass Blue arrow: increased haemoperitoneum

**Figure 4:**
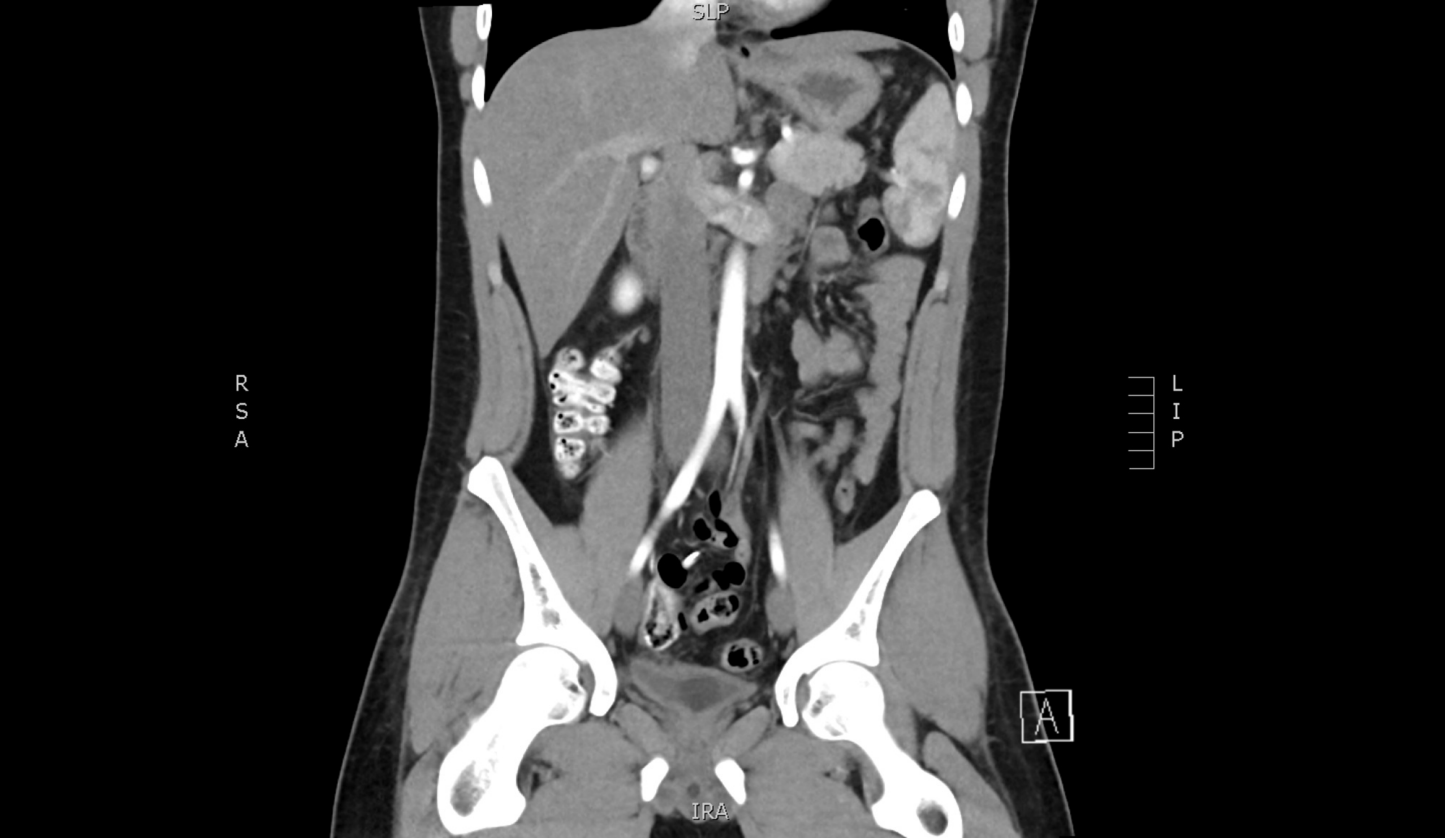
Computed tomographic angiogram on postoperative Day 2 with no blush or haemoperitoneum

A 20-year-old male presented to our emergency department with a one-day history of worsening central and epigastric abdominal pain associated with multiple instances of vomiting. He denied any intake, recent trauma, or any recent infective symptoms. He also complained of chest pain, shortness of breath, and lightheadedness. Our patient was a fit and well 20-year-old male with no significant medical or family history. He had a systolic blood pressure between 100 - 140 mmHg, was tachycardic between 100 - 115 beats per minute (bpm), and required increasing amounts of opiate analgesia. His haemoglobin was 129 g/l and the lipase level was within normal limits. Computed tomography (CT) scan showed moderate free fluid throughout the abdomen, particularly within the pelvis, of an intermediate density of 35 - 40 Hounsfield units and a lobulated heterogenous mass extending from the greater curvature of the stomach into the greater omentum measuring 11.7 x 6.3 x 13.7 cm, in keeping with a neoplasm, such as a gastrointestinal stromal tumour (Figures 1-2). A follow-up with a CT angiogram was performed to consider treatment with angioembolisation if a blush was present. This scan showed caudal migration of the abdominal mass, as well as an interval increase in haemoperitoneum, but no evidence of an active arterial haemorrhage (Figure 3). These findings were initially thought to be a bleeding neoplastic mass that had ruptured from its pedicle. Our patient became acutely tachypnoeic and peritonitic. The decision was made to perform an emergency diagnostic laparoscopy. The findings of the operation were 3 litres of haemoperitoneum with a large pelvic haematoma which corresponded to the heterogeneous mass seen on preoperative CT; however, no bleeding source was found. There was also a small haematoma in the lesser sac over the proximal greater curve of the stomach. The haematoma and blood from all four quadrants and the lesser sac were evacuated, and drains were inserted. The patient remained haemodynamically stable postoperatively; his haemoglobin had dropped from 129 g/L preoperatively to 87 g/L on Day 1 postoperatively but remained stable over the course of his recovery. A CT angiogram was repeated Day 2 postoperatively which confirmed no ongoing haemorrhage (Figure 4). His recovery was uncomplicated, and further investigations to date with a consultation with the vascular medicine physicians have not identified the underlying cause of the spontaneous intraperitoneal haemorrhage. Further investigations will include a CT angiogram of his head and chest to exclude aneurysmal conditions. Our patient was discharged home on postoperative Day 5.

## Discussion

Intraabdominal haemorrhage can occur from many different causes, including trauma, iatrogenic, ruptured aneurysm, gynaecological conditions, malignancy, and inflammatory or autoimmune processes. Spontaneous or idiopathic causes are much rarer. Cases of abdominal apoplexy are usually described in the context of underlying vascular disease, most commonly atherosclerotic disease and hypertension [3]. Coagulopathy seems to be another risk factor with several reported cases of abdominal apoplexy occurring in cirrhotic liver disease, hemophilia, and Factor V Leiden deficiency [2, 4-6]. Cases have also been reported in the setting of autoimmune conditions, such as alpha-1-antitrypsin, polyarteritis nodosa, and rheumatoid arthritis [7].

There is a 2 to 3:1 male preponderance, typically presenting between 50 - 70 years of age. The presentation usually includes abdominal pain, which may be associated with nausea, vomiting, and diarrhoea. The condition is clinically difficult to differentiate from peptic ulcer disease or acute pancreatitis [1]. Progression of symptoms relates to the rate of the bleed with worsening pain and development of haemodynamic compromise [8].

When initially described, the diagnosis was made during laparotomy. Diagnostic methods have evolved with easier access to imaging. CT is considered the most important imaging technique with CT angiography proving useful to identify the sites of active bleeding [8]. Focused assessment by sonography in trauma (FAST) can also be useful in the haemodynamically unstable patient. Bleeding vessels are typically found at the secondary or tertiary branch points from the aorta. The most common sources of bleeding are the middle colic, left gastric, and haematoma of the mesentery [9], although a bleeding vessel has been found in only 62% of explorations. Typically, identification and ligation of the bleeding vessel are performed via exploratory laparotomy; only one other reported case has been completed laparoscopically [10]. With non-operative management, mortality from abdominal apoplexy has been reported to be nearly 100%. Explorations which do not identify a bleeding source are associated with a 40% mortality, which is reduced to 8.6% if the bleeding source is ligated. Postoperative angiography is often performed in non-therapeutic explorations because of the high risk of mortality from rebleeding [11].

Our case report presents a unique case of abdominal apoplexy. While the typical patient is in their fifth or sixth decade of life with known systemic conditions associated with vasculopathy or coagulopathy, our patient was a young and healthy person with no known medical conditions or genetic predispositions for vasculopathy. While the patient showed signs of deterioration, conditions were deemed appropriate for an exploratory laparoscopy and evacuation of the haematoma, with a low threshold for converting to laparotomy. While no bleeding source was found, the absence of an ongoing bleed was excluded with a period of observation and follow-up CT angiogram two days postoperatively. Furthermore, the diagnosis was made more challenging due to the ambiguous findings on CT suggestive of a haemorrhage from a ruptured neoplastic mass. We recognise that this was a rare case of spontaneous intraperitoneal haemorrhage and have consulted vascular medicine to exclude underlying conditions that may predispose our patient from subsequent episodes.

## Conclusions

Abdominal apoplexy is a fatal condition which can progress rapidly. Better awareness of the condition may help to improve survival by promoting early diagnosis and intervention. Our case report highlights that, while most cases of abdominal apoplexy occur in patients in the fifth or sixth decade of life with systemic conditions that can precipitate spontaneous vascular injury, it can also occur in young patients who have no preexisting conditions. This case also shows that in the appropriate clinical setting, exploratory laparoscopy and evacuation of the haematoma is a safe and viable option in the management of abdominal apoplexy.
